# Types of statistical arguments in empirical research in psychology

**DOI:** 10.1098/rsos.260798

**Published:** 2026-10-28

**Authors:** Gustavo Andres Marquez Flores, Rink Hoekstra, Henk A. L. Kiers, Don van Ravenzwaaij

**Affiliations:** ^1^ Psychometrics & Statistics, University of Groningen, Groningen, The Netherlands; ^2^ Educational Sciences, University of Groningen, Groningen, The Netherlands

**Keywords:** statistical arguments, argumentation, statistical reasoning, typology, inductive coding, metascience, meta-research

## Abstract

Numerous solutions have been proposed to address the problem of statistical misuse and misunderstanding in research in psychology. This might have diverted attention from how or whether scientists are actually engaging in statistical reasoning by arguing about and with statistics within the context of the reasoning involved in empirical research in their written papers. In this paper, we studied statistical reasonings in psychological research papers. Specifically, we inductively coded arguments of 42 papers from seven highly cited psychology journals. With these data, we formed a partial, empirical typology with which we described different types of statistical arguments used in the literature. Papers, and their statistical arguments, in empirical psychology can be interpreted as possessing a ‘legal’ nature. Common arguments, such as statistical significance, are often used as a tacit and concise way of certifying that a claim, including conclusions, is empirically and statistically substantiated under the social standards of good research in our field. However, approval of research based on certifications may hinder the exposition and clarity of statistical arguments.

## 1. Introduction

How do researchers bridge the gap from formal statistical results to conclusions about their research questions? In what has been termed statistical reasoning, researchers ‘reason with statistical ideas and make sense of statistical information’ [1, p. 1]. Attempts to understand this process have mostly focused on misuse and misunderstanding of statistics [2], especially of statistical concepts strongly connected to inference [3]. However, we know surprisingly little about empirical researchers’ statistical reasoning aside from what we consider faulty or skewed. In this study, we attempt to learn more about how researchers employ statistical reasoning in empirical research.

In 1942, Joseph Berkson [4] pointed out a problem with making inferences with certainty using null hypothesis significance testing (NHST). It is impossible to absolutely disprove the null hypothesis when testing merely indicates that the difference between two groups is unlikely to be derived from a population difference of zero. This implies that one must always address the possibility that chance brought about the results of an experiment. Furthermore, researchers neither know the probability of their result being a false positive after rejecting the null hypothesis [5,6] nor the probability of the null hypothesis itself [7]. This uncertainty, however, is not always addressed in empirical research [8].

In addition to researchers, editors and reviewers also seem to expect significance (*p* < 0.05) to mean much more than what it really does [9–11]. This culture [12,13] of seeking significant results based on misinterpretations or false expectations seems to ensue in a high frequency of *p*-values below 0.05 [14] and questionable research practices [15] and has led to unsuccessful attempts to replicate initially considered robust findings in psychology [16]; sparkling reform movements that either sought to instaurate estimation instead of NHST [17] or a Bayesian approach instead of a frequentist framework [18] to improve empirical research. However, problems of expectation and interpretation are also found when people make estimations using confidence intervals (CIs) [19] or when they test hypotheses with Bayes factors [20,21].

Although the work on identifying statistical reporting errors is prolific, less is known about what statistical thinking and, thus, reasoning look like [1,22,23], let alone in written format. There is no descriptive account of how researchers make inferences and interpret statistical results by combining ideas about data and chance in their reports [1]. Statistics is a deductive, logical structure from which conclusions can be drawn [24]; therefore, when using it, researchers must present in-depth arguments that validate and contextualize the inferences that follow this structure. When drawing conclusions, they must understand how variation, uncertainty, sampling process, data collection, unobservable variables’ impact, measurement and bias interact when carrying out an analysis [1,22]. Considering how hard it is to formulate a valid statistical inference, we would expect that researchers deal with complex and challenging ideas and information, especially while writing their final report. Consequently, their paper should show such thought complexity, for researchers are making a sensible case for their conclusion by laying out the arguments for and/or against it.

Six years after Berkson's article [4], Dorothy Sayers highlighted two shortcomings that hampered argumentative discussions and comprehension during their time. Firstly, Sayers [25] criticized writers of her day for failing to define their terms when arguing. Above, we alluded to erroneous definitions of statistical concepts that can undermine the conclusions reached by researchers. Secondly, Sayers [25] claimed that mathematics teaching was disjoint from logic, so students believed that they were unrelated. Chris Wild made a very similar remark about education a few decades ago. ‘We do not want to develop graduates who keep what they know about psychology … and what they know about statistics in separate mental compartments’ [26, p. 170]. We do not know whether logic, psychology and statistics are segregated by ‘water-tight bulkheads’ [25, p. 4] when learned nowadays. Nevertheless, by inspecting statistical reasonings through arguments in written articles, we should be able to observe how connected and disconnected these three are.

In this project, under the larger umbrella of reasoning, we consider arguing as offering a set of reasons for or against a conclusion [27]. We undertake a qualitative approach to identify the scope of statistical reasoning used in articles published in prominent psychology journals. Thus, we would like to describe the set and structure of reasons that psychologists give to support or undermine a scientific inference. For this aim, we intend to identify the different arguments researchers lay out with and about statistical information stemming from their results through inductive content analysis [28] and the construction of an empirically grounded partial typology [29,30]. To this end, we have collected data on and about the statistical results of an article's first inferential claim, which should be in the sections of results and discussion [31]. We are making two assumptions throughout our work. First, we assume that authors write with intention, so we are not carving out what we think authors wanted, tried or subconsciously attempted to convey. Rather, we attempt to stay as close as possible to what the authors communicated in writing [32,33].

Our second assumption is that when someone argues in a research article, said argument can be identified by analysing the principled relationships between propositions, which are assertions or statements about something. Connecting words, like ‘despite’, ‘because’ and ‘for’, should aid us in identifying such principled relationships [34]. Our overall purpose is to provide a description of written statistical arguments indicated by data [35], which should shed light on relevant aspects that could explain how people think and reason about and with statistics in the larger context of scientific reasoning when reporting empirical research.

## 2. Method

### 2.1. Reflexivity statement

The authors of this paper have a sceptical attitude towards the typical use of statistical inference in general and NHST in particular and have explicitly written about these concerns. Undoubtedly, this has coloured our interpretation of the outcomes of the present investigation and has influenced the approach that we have taken. Finally, the first author has some familiarity with theological literature, including a biblical study method, which has influenced the approach that we have taken.

### 2.2. Design

For the present study, we followed a qualitative research design. On the qualitative research continuum, our study leaned more towards tightly rather than loosely structured research designs [30,36]. Decisions on research questions, sample characteristics and coding style were made before collecting data owing to time constraints, practicality and the nature of our investigation. Still, we remained flexible in altering our research design when necessary.

Accordingly, we analysed arguments’ premises (the reasons for) and conclusions through their syntactic composition [34,37,38]. Subsequently, we inductively coded [39] and aggregated these arguments according to patterns similarly to how it is done in conventional content analysis [28]. However, when we were gathering data, we chose to make a partial typology [29,30] based on an argumentation layout framework [40]. We did so because we believed a typology gave a better account of the written arguments in comparison to aggregating them by pattern similarity. You can view the process of our study in greater detail in figure 1.

### 2.3. Sampling strategy

We gathered data from English-written psychology papers that reported at least one quantitative analysis on empirical data. We have included meta-analysis and empirical studies that had at least one inferential statistical analysis. Descriptive articles that made such a claim were also included. We ruled out articles about statistical procedures dealing with mathematical facts, simulation or psychometric studies.

Our sample is from journals that have a high journal impact factor (JIF), *H*-index (*H*) and CiteScore (CS), which are metrics of journals’ influence that use citations for calculation. Thus, articles published in these journals are, on average, frequently read and referenced. The measurements for the JIF were gathered from [41], for the *H*-index from [42] and for the CS from [43]. In the SCImago Lab and Elsevier websites, we selected the subject area of ‘Psychology’ and the filter of ‘Journals’ for our search. Then, we did a quick inspection of the 50 psychology journals with the highest CS score up to the latest available measurement – 2023. We did the same for the 40 psychology journals with the highest *H*-index up to 2024. We selected journals based on their description, excluding those that published mostly review, theoretical and methodological articles. We also excluded journals in which psychology articles were published occasionally on a quick inspection of their latest volume. Finally, we also based our selection on comparing and selecting the highest JIF possible between journals. The six chosen journals are as follows:

— *American Psychologist* (JIF = 12.4; *H* = 283; CS = 18.5)
— *Journal of Applied Psychology* (JIF = 9.4; *H* = 353; CS = 17.8)
— *Psychological Bulletin* (JIF = 17.3; *H* = 379; CS = 33.6)
— *Psychotherapy and Psychosomatics* (JIF = 16.3; *H* = 123; CS = 29.4)
— *Nature Human Behaviour* (JIF = 22.3; *H* = 113; CS = 36.8)^1^
— *Cognition* (JIF = 2.8; *H* = 235; CS = 6.2)

*Cognition* was chosen because the previously selected cognitive psychology journal published few empirical articles. Next, we selected seven papers published during 2025 from each journal. It was within our original plan to extend our sample beyond 42 papers if we had not reached saturation by then. Nevertheless, we consider that increasing our sample would not bring new or informative data [30] that would change our interpretation at its core. We selected the journals’ issues published between April and June of 2025. A list of exclusions can be viewed in appendix A in the supplementary material, and our dataset can be found in our OSF repository, together with the preregistration of our study.

### 2.4. Data analysis

The first author read and collected data from each article. We identified (i) each article's first mentioned inferential claim. Then, we gathered (ii) its corresponding statistical result and also collected (iii) the ensuing discussion and/or conclusion. Here the units of analysis [44] were the sentence(s) or paragraph(s) in which authors argued about how their result(s) supported or undermined a conclusion(s) about their main hypothesis. Accordingly, we did not only collect arguments using statistical results but also arguments about said statistical results. That is, premises that were used to support the inference made with a statistical result, for example ‘our results replicate previous findings’. Even so, we excluded propositions that were too far removed from a statistical result and its ensuing claim, like those more concerned with theory construction.

To support argument recognition, we used some elements of a theological method called arcing [34,37,38]. The aim of this method is to provide a ‘list of possible logical relationships, with descriptive names’ [38, p. 10] to aid text interpretation. For example, a keyword like ‘*because*’ signals that, frequently, following ‘*because*’ we find the argument that supports the statement before said keyword. This is a ground subordinate relationship, for the second clause functions as the ‘ground’ of the first one [34,37,38]. Sometimes writers do not explicitly use connecting keywords between sentences. However, for instance, understanding that one distinct statement can function as the ‘ground’ of the previous proposition will help us to detect arguments that are not explicitly spelled out. Appendix B in the supplementary material presents some logical relationships employed in this method called arcing. In summary, we started off by looking at the syntax of sentences and paragraphs to examine potential logical relationships between clauses. Then, we looked for patterns or similarities between logical relationships to codify our data.

Our codification was inductive [28,39]. We cyclically collected and analysed units of analysis, creating codes that could be removed, confirmed or modified by new data input later. As we were grouping codes with similar arguments, we realized that we could create types of arguments based on a combination of attributes [29,30]. To construct this typological system, we used Toulmin's argumentation model [40]. Argumentation is ‘a communicative and interactional act complex aimed at resolving a difference of opinion with the addressee by putting forward a constellation of propositions the arguer can be held accountable for to make the standpoint at issue acceptable to a rational judge who judges reasonably’ [45, p. 2102].

The gist of argumentation theory is to ‘provide adequate instruments for analysing, evaluating and producing argumentative discourse’ [45, p. 2104]. We availed ourselves of one of these models because we considered that arguments in papers were presented to be judged by rational judges who were tasked with evaluating how cogent the reasons for a standpoint or conclusion were [45]. In fact, the model that we are using [40] also showed examples with keywords that are similar to the ones used in arcing. Because of that similarity, it was simpler to organize our codes into types based on the model as we describe next.

#### 2.4.1. Our typology's attributes and Toulmin's argumentation model

We grouped our collected data in a typology based on three attributes: (i) result claims, (ii) presuppositions, and (iii) argumentation components. First, result claims were propositions that revolved around or referred to the statistical results of a study. In the majority of cases, these were conclusions. Said claims were further divided according to whether they were consonant or dissonant with authors’ expectations.

Expectations in the context of our research could be indicated through a hypothesis, a theoretical statement or a prediction (i.e. finding a large instead of a small effect size). Thus, a consonant result claim was defined as a statistical result that was consistent with or followed any sort of expectation, regardless of whether authors later qualified said consonant claim with a limitation. For instance, a result that is not generalizable to other populations but that supports the hypothesis is considered consonant. Such a claim, however, would be considered dissonant if it does not follow the expectation set by a hypothesis, regardless of whether authors later accepted and qualified it with a set of limitations. For example, authors may conclude that a treatment worked for a subgroup of women because the corresponding result was the only statistically significant one of their study, even when their expectation was that the psychotherapy would be effective for everyone. That would be a dissonant result claim even if authors later stated that a subgroup result should be taken with caution.

Secondly, analogous to our approach with consonant and dissonant result claims, we divided the attribute of presuppositions into two extremes: credible and contested. Pieces of text in the credible presuppositions category refer to authors stating that some presuppositions they had were, reasonably, upheld. Presuppositions here could be arguments for why the results answered the research question or arguments for why results had more credence because of some authority (i.e. a respected paper or expert) or an employed statistical technique. All of these have the purpose of arguing for why the results of a study should be considered as trustworthy. The category of contested presuppositions was for when authors considered that the alluded properties of a trustworthy study and its results could be called into question. Then we categorized texts arguing for or against these credited or contested presuppositions.

We were inspired by Stephen Toulmin's [40] model of argumentation (see figure 2) for the third attribute: argumentation component. We considered that there are usually three ways in which authors lay out their argumentation in papers: inferences, backings and rebuttals. This is different from the original model, which included six components.

**Figure 1.**
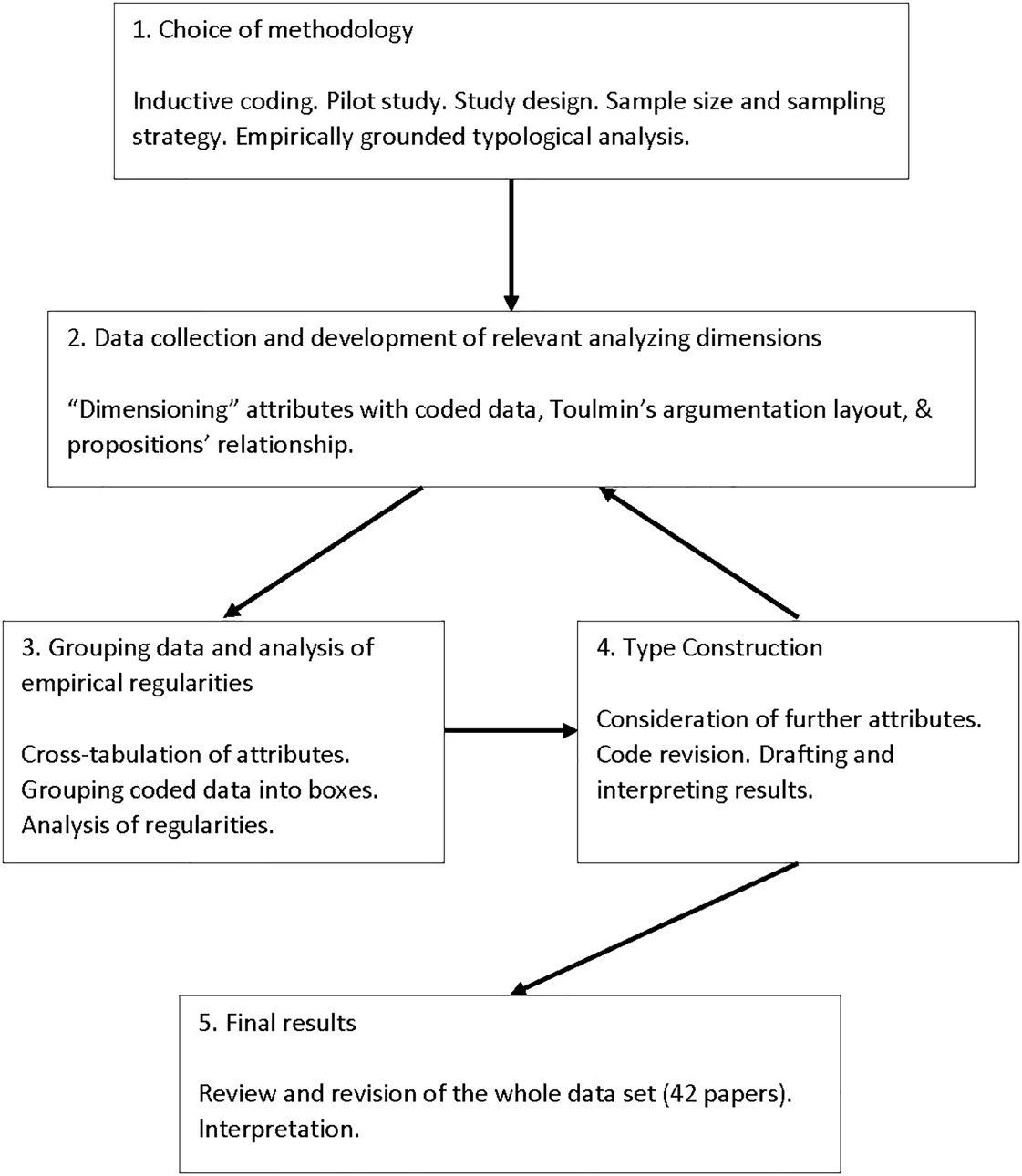
Procedure flowchart. We started off with choosing the methodology we believed would better match our research question. As depicted, data collection, analysis and interpretation were cyclical processes until the 42 papers were examined.

**Figure 2.**
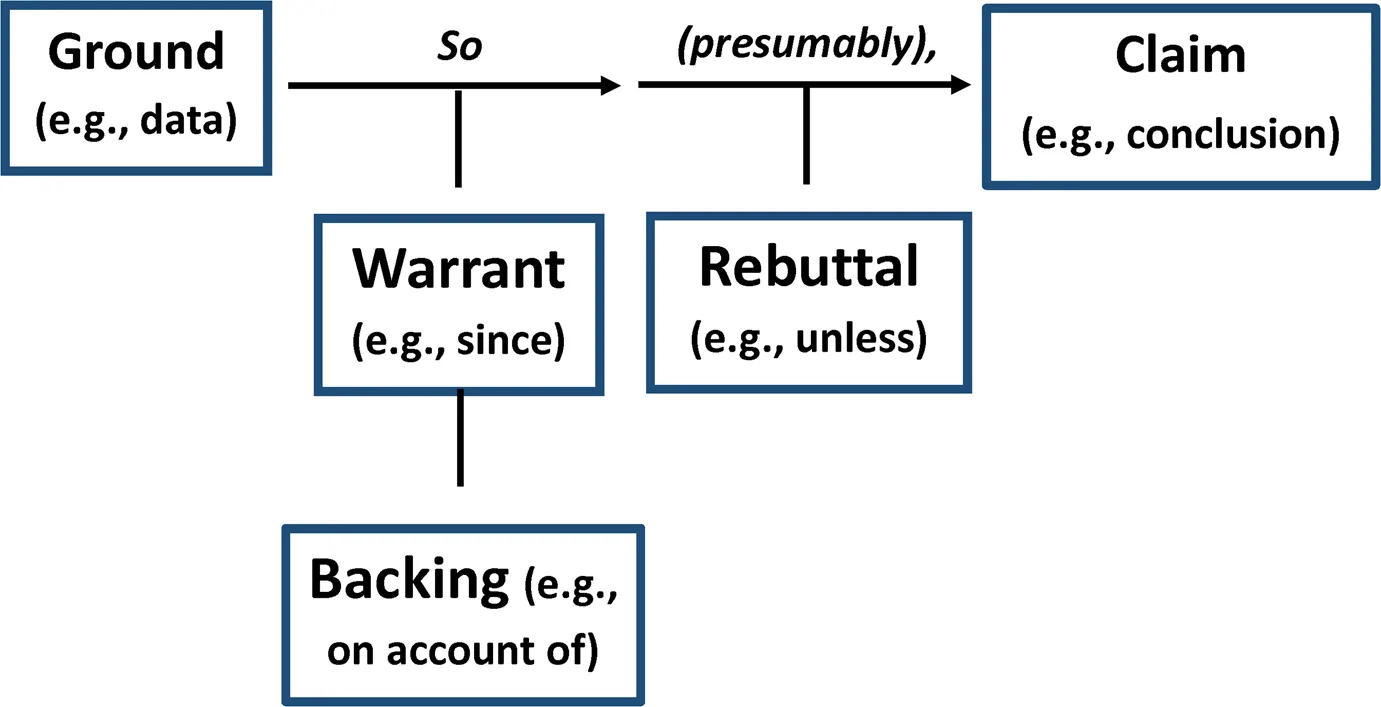
Toulmin's model of argumentation adapted. The ground, warrant, backing, rebuttal, qualifier and claim are the model components. The other words and phrases – so, since, unless and on account of – are keywords that Toulmin used to describe each component. We found them similar to how keywords are used in arcing. In the image, the word presumably is representing the qualifier; therefore, other words that could be placed there are certainly, possibly, may, might and others.

To begin with, the first component of the original model is the data—from here on ground (i) to avoid confusion. The ground is the information or facts that function as the foundation upon which the arguer seeks to establish a proposition, that is a claim (ii), which is the second original component. The ground could take the form of what traditionally is known as the minor premise, while the claim would be the conclusion. Toulmin's [40] example of a ground is ‘Harry was born in Bermuda’ to which the claim is ‘Harry is a British citizen’.

The third component, the warrant (iii), is the bridge that takes one from the ground to the claim. That is, it is a rule, principle or anything else that legitimizes the inference by showing that the ground has a bearing on the claim. It can take the form of what is usually known as the major premise: ‘if p, then q’. The warrant of Toulmin's example [40] is ‘a man born in Bermuda will generally be a British citizen’. The rebuttal (iv) applies to those circumstances or conditions in which the warrant no longer works, while a qualifier (v) – ‘certainly’, ‘may’, ‘presumably’ etc. – indicates the strength of the warrant once the rebuttals have been weighed. One rebuttal that may refute our warrant is that ‘Harry has changed his nationality’ or that ‘both of his parents are extraterrestrials’ [40]. Therefore, because we do not have assurances about both of these rebuttals, we would have to add a qualifier like presumably to our claim: ‘so presumably he is a British person/citizen’.

Finally, there is the backing (vi) of the warrant, which sustains the soundness of the argument. Arguers back their warrants with other assurances like facts or theoretical considerations that grant them authority or currency in the subject under discussion. So, if Toulmin's [40] warrant about Harry, ‘a man born in Bermuda will generally be a British citizen’, would be challenged, he could back this warrant by citing the specific statutes of the British Nationality Act. Of course, the backing here is about how Harry is seen in the eyes of the law. Not all backing can be done with the law. For instance, backing a warrant such as that a whale may be taken to be a mammal by means of a law would be nonsensical. Instead, such warrants are backed by a scientifically accepted taxonomy. Thus, there is a field-dependency [40] when it comes to the needed backings to establish warrants.

Coming back to the three components we used in our research, we grouped those propositions in the backing (i) component, the meaning of which seemed to support the legitimacy of any principle that authors follow to move from a piece of information to a claim. The inference (ii) component refers to texts written in such a way that we could verify that the movement from ground to claim had been done [46]. An inference, thus, could contain three to four of Toulmin's components: ground, claim, warrant and, from time to time, backing. In our data, the inference had a claim that was usually explicit, a ground that was often explicit and a warrant that was never explicit. Authors commonly mentioned the backing instead of the claim. We can take another example from Toulmin [40, pp. 101–102] to show what we mean by this:

*Ground: Petersen is a Swede;*

*Backing: The proportion of Swedes who are Roman Catholic is less than 2%*

*Warrant: A Swede can be taken almost certainly not to be a Roman Catholic;*

*Claim: So, almost certainly, Petersen is not a Roman Catholic.*

People could use an abridged version of this argument in daily life by saying that the proportion of Roman Catholic Swedes is less than 2% (this is the backing in Toulmin's model), so, almost certainly, Petersen is not Roman Catholic because he is Swedish (the conclusion). Rebuttal (iii), the third type of argumentation component, was for when authors acknowledged or argued about instances or conditions by which their result claims would no longer be valid. We stress that we used Toulmin's model as a guide to create our typology rather than a strict theoretical framework to be followed.

## 3. Results

### 3.1. Partial typology of arguments

The final combination of the typological attributes that we described in our methods can be seen in table 1. It shows the types of statistical arguments in empirical research in psychology that we have constructed with our data by combining such attributes. These types will further be described below, each accompanied by two example quotations. It is worth noting that after examining all the included papers, we scarcely found instances in which written argumentation resembled the complete, integrated layout of Toulmin's model for argumentation. As we will see, the components are rather fragmented and isolated, following the traditional format for articles in psychology [31], which encourages segregating introduction, methods, results and discussion into different sections. In addition, we have included other quotations to each type of argument in appendix C in the supplementary material.

**Table 1.** Theoretically conceivable combination of attributes.

argumentation components	result claim		presupposition	
	consonant	dissonant	credible	contested
backing	a. literature b. consistency of sensitivity analyses c. study characteristics d. explanation	a. despite small, the effect is important b. literature	a. avoiding type III errors b. literature c. design d. statistical bedrock	
inference	a. accepting the null hypothesis b. (not-)rejecting the null hypothesis c. estimation d. description e. regularity f. the study g. suggestive results	a. embracing unexpected results b. despite significant, it is meaningless		
rebuttal	a. credibility b. external validity c. non-experimental design d. design e. future research	a. despite null, another one is significant b. credibility		a. design b. sensitivity analysis c. literature d. inconsequential

*Note:* This is a partial typology because not all combinations of attributes (cells) were filled even though merger was theoretically conceivable.

### 3.2. Backings

#### 3.2.1. Backing consonant result claims

The first type of backing that we distinguished in our data for consonant result claims was obtaining or having results that resembled those registered in the literature as a precedent. How and in what way was usually not very clear:^2^

P22: ‘This result **aligns with existing literature** on therapeutic interventions for …’

P27: ‘Our findings focusing on Europe **mirror** somewhat those of Surkalim *et al.* (2022).’

A second type of backing was when sensitivity results were used to support the main result's validity. In the legal setting, we could think about them as assurances that the results were not circumstantial owing to statistical artefacts:

P8: ‘Importantly, all relationships **remained significant after controlling for** previously examined moderators (leader benevolence and power distance orientation) and alternative positive and negative states’

P36: ‘These results were **supported by the analyses** on pain unpleasantness ratings (see APPENDIX A: Supplementary material 3).’

Thirdly, authors thought that preregistration, methodology, type of statistical analysis, design or any other characteristic of their study backed how they got their inference. These characteristics have some value for the research community to which authors appeal for assurances about their inferences. How valuable they are and why was not usually made explicit in the papers:

P1: ‘**With this comprehensive multilevel, multivariate meta-analysis**, we set out to shed light on the question …’

P10: ‘In **the first preregistered hypothesis** it was predicted that the investigated conditions would … This hypothesis was confirmed…’

Finally, authors further clarify or define their results in theoretical or methodological ways. After all, a reader might not be convinced without understanding what the author is talking about. Hence, these explanations function as rhetorical devices to give assurances to the reader that the result is coherent or does make sense in light of theoretical predictions, methodology or reasonableness in general:

P9: ‘There was very little difference in the risk associated with moderate or high-memory specificity, **consistent with the theory** (Williams *et al.*, 2007) that mental illness only arises if …’

P21: ‘These day-specific changes align with the structured design of B4DT, **in which each day of the treatment is intended to** target different psychological processes.’

#### 3.2.2. Backing dissonant result claims

Alternatively, authors use a few ways of backing dissonant result claims, although this happens less frequently than the backing of consonant result claims. In three articles, researchers saw the need to provide assurances that what they found was relevant to their final claim despite some other features that indicated the opposite. For instance, when authors thought that a small effect size did not follow initial expectations, they gave assurances about their result by indicating that it was, for example, either statistically significant or clinically meaningful. In other words, authors emphasize a specific feature of the result. In table 1, we called this type of argument despite small, the effect is important:

P11: ‘**Although overall effect sizes were small-to-moderate, small effects are clinically meaningful** when accumulating over time (Funder & Ozer, 2019).’

P12: ‘**Small but significant** differences across levels of cognitive demand (p = .005) emerged for chronic studies’

In other instances, inferences were dissonant with their hypotheses, theories or expectations, so authors pointed out the existence of literature or alternative antecedents that could provide assurances:

P27: ‘Our findings for midlife in the United States contrast with those of historical declines and stability observed in … (Clark *et al.*, 2015; Hawkley *et al.*, 2019; Mund *et al.*, 2020) **but are similar to** … (Buecker *et al.*, 2021)’

P34: ‘This finding **is in line with other international studies** on trust in scientists 5–7. Our study thus confirms, expands and strengthens previous work that refutes the narrative of a wide-ranging crisis of trust’

#### 3.2.3. Backing credible presuppositions

Authors also gave assurances to the choices they made in their research. Avoiding type III errors in table 1 refers to when authors justified conducting an analysis or the overall study in a certain way to avoid ‘giving the right answer to the wrong problem’ [47, p. 134]. Thus, they used the objective of their study as a justification for making certain choices:

P7: ‘**To test our specific hypotheses, we intentionally chose not to include** contextual information …’

P31: ‘… the included male-specific observations are narrowly focused on intimate partner physical violence against men **to reflect this study's emphasis on** gender-based physical violence’

Researchers also backed their choices by stating that they were following literature in the form of expert opinions or previous designs:

P8: ‘We then **followed Aiken and West's (1991) suggestion to** plot the interaction effects at high (+1 SD) and low (−1 SD) levels of event disruption …’

P13: ‘**Following previous meta-analyses** (Dai *et al.*, 2023; Donald *et al.*, 2021; Macnamara & Burgoyne, 2023), we assessed study quality based on four indicators …’

In a few cases, we found researchers trying to give assurances about the strength of the design or study overall:

P12: ‘It is important to note that the adult population (n = 127) **was equally represented** compared to children and adolescents (n = 112)’

P24: ‘**The primary strengths of this trial** include its robust RCT design, in which we rated adherence to both treatment manuals, included long-term follow-up and conducted the trial in real-world clinical settings.’

Finally, authors backed a result with what we called statistical bedrock (see table 1); they relied on a statistical result or analysis that would give them assurances about trusting the validity of their results:

P9: ‘Low heterogeneity supports the consistency across studies and **lends confidence** to results.’

P16: ‘Throughout the sequential model specifications, the ALD models that included a cubic term **always fit the data better**, providing some evidence of nonlinear SEC growth patterns as students move through school.’

### 3.3. Inferences

#### 3.3.1. Inferences and consonant result claims

Researchers made inferences to support claims in empirical research in multiple ways, logically, since the set of statistical techniques and approaches to take from is broad. There are, overall, two ways in which researchers argue for their final claim after making an inference. Reasonings that did not fall into these two groups were placed in the type labelled ‘other’ (see table 1). The first group encompasses researchers directly addressing their statistical information. This group consists of reasonings in which researchers wind up accepting the null hypothesis of no effect based on a null result (not necessarily through significance testing) or accepting the difference between a null result and another significant result as a significant difference:

P2: ‘… the relationship was strong and significant for liberal participants (slope = −0.64, p < 0.001) but nonsignificant for conservative participants (slope = −.21, ns) … as I predicted, only liberal employees tend to view their politically dissimilar coworkers as lower in person trustworthiness; **the pattern does not appear among conservatives**.’

P39: ‘There was **no quadratic effect** in either episodic (age-squared beta mean: -0.04; HDI: [− 0.13; 0.04]) or semantic tasks (age-squared beta mean: 0.03; HDI: [− 0.05; 0.11])’

Researchers also rejected or did not reject the null hypothesis in favour of the alternative hypothesis using significance testing. The previous type of arguments exclusively claimed support for the null hypothesis, while here the claims were made more in accordance with the logic of NHST. Consequently, the null hypothesis could either be rejected or not, which falls more in line with how NHST is recommended to be used:

P3: ‘… **the relationship was significant** (γ02 − γ60 = −.56, p < .001; Model 3; table 4). Hypothesis 1 was thus supported.’

P6: ‘I also found **a significant positive relationship** between experimentation T4 and BM adaptation T5 (b = .172, SE = .052; p = .001).’

Some forms of estimation were also present in our dataset. We included in this type those inferences for which we did not find an explicit statement about the null hypothesis:

P14: ‘**The largest effects were yielded by treatments for** OCD, PTSD, and specific phobias (g = 1.18–1.25; NNT: 4–3).’

P26: ‘Insomnia was the only physical diagnosis associated with **an increased probability** of seeing a specialist (AME = 0.023, CI [0.004, 0.042]).’

The final type of argument in the group of inferences made by directly addressing the statistical result is based on descriptive results only since claims were based on either figures or statistical estimates that did not have measurements of uncertainty:

P16: ‘**As indicated by**figure 1, results for GM generally showed increases in scores over time…’

P21: ‘First, the high rates of adherence to ERP strategies, **with compliance above 70% on day 2 and above 80% on day 3**, indicate that patients generally maintained a strong commitment to the therapeutic tasks throughout the intervention.’

The second group of reasonings is that in which the statistical inference is not directly addressed but alluded to. Firstly, there were arguments about regularity. Namely, researchers used the consistency of multiple analyses, results, experiments and/or studies to give support to a claim or conclusion. Note that we differentiate these from arguments about regularity based on previous results in the literature (see §3.2.1). Again, how consistency got defined was unclear. We identified these arguments when multiplicity was used as a whole to support a claim:

P5: ‘In Study 1, while using supervisors’ reports of higher level managers’ leadership styles provided a separate rating source that helps to reduce common methods bias, those ratings may not align with employees’ perceptions of the managers. Yet, **consistent results from Study 3 boost confidence in our findings**.’

P10: ‘This hypothesis was confirmed by **the statistical analyses that clearly showed** that the overall case–control effect was not independent of diagnoses.’

We have seen above, in the backing sections, that consistency was also used to give assurances about a result in the case of sensitivity analyses or relying on antecedents in the form of previous results. Therefore, results could be used in arguments in multiple ways. Next, the study, project or research is sometimes used to make a claim, without making specific which attributes (e.g. the statistical results, the design, characteristics of the sample and all of the above) of said study lend credence to the claim:

P26: ‘**This study provides a foundational understanding** of care delivered by prescribing psychologists versus other health care providers’

P34: ‘**Our study thus confirms, expands and strengthens** previous work that refutes the narrative of a wide-ranging crisis of trust.’

Finally, researchers integrated both their statistical results and their limitations into one somewhat qualified final claim. We call this type of argument suggestive results:

P10: ‘Although our results do not provide causal and mechanistic insight, **they nonetheless suggest that** atypical hand preferences are …’

P15: ‘… despite these findings, we should consider this data set highly heterogeneous, which may indicate underlying differences between trials that we did not identify. The results should **be considered with caution**.’

#### 3.3.2 Inferences and dissonant result claims

When results did not follow some sort of expectation, researchers usually embraced them, sometimes attempting to make inferences or explanations about them:

P16: ‘… by age 12, students typically realize that sometimes ability can limit outcomes, regardless of the amount of effort. Therefore, **it is surprising that** GM continues to rise through adolescence.’

P17: ‘**The first striking result of the experiment** was that no strong support for the main effects of a powerful messenger was found, as shown in Rows 5–8. This result is in sharp contrast to the results of previous studies …’

In two papers, we found instances in which researchers got a result that could be in line with their expectations, but they had to deal with some other feature that indicated the contrary. Since this last feature had a larger weight for them, they wound up concluding what was against their expectations (in table 1, despite being significant, it is meaningless):

P30: ‘… effect sizes were positive, statistically significant **but equivalent** (that is, they fell within the preregistered SESOIs). This indicates a difference that is too small to be theoretically meaningful between adolescents with and without a mental health condition.’

P31: ‘We identified an association between physical GBV and seven health outcomes … **although these associations and/or their underlying evidence were rated as weak**, receiving one- and two-star ratings in the Burden of Proof framework.’

### 3.4. Rebuttal

#### 3.4.1. Rebuttals of consonant result claims

The most typical example of arguments in this category is authors raising concerns about the credibility of their results and claims. That is, although their results had met expectations, they mentioned, first, possible alternative conditions for this to happen that could undermine making a knowledge claim out of those results. Most commonly, this was done by mentioning potential confounders, sample size, type of control group or omitted variables. Researchers also sometimes argued that even though the (main) result was consonant with expectations like a large effect size, there was a characteristic of it or the result of a sensitivity analysis that contradicted it, like a non-significant result (see credibility in table 1):

P6: ‘Conducted at the start of the pandemic, it employed a program for entrepreneurs, which **may have influenced** my findings.’

P11: ‘However, longitudinal studies can still be subject to confounding. It is possible that **a third variable explains both** socioemotional problems and screen use.’

External validity was one of the rebuttal arguments that authors used to nuance claims based on their studies:

P23: ‘… the lack of gender diversity in our sample, predominantly consisting of females, **restricts the generalizability** of our findings to other genders.’

P25: ‘It is unclear if our findings **apply to** clinical samples with more comorbidity.’

Authors were also concerned with the caveats that non-experimental research designs impose on causal claims:

P13: ‘… we emphasize that meta-analysis is primarily a correlational method, particularly concerning the moderator analyses. Hence, the results reported in our meta-analysis **do not indicate** causal relations.’

P18: ‘A significant limitation of our study is that we rely on observational data, and we are therefore **unable to make any strong** causal claims.’

Researchers pointed out study limitations in the form of flaws or better alternatives to the design that would have improved the insight of the studied phenomenon:

P19: ‘We posit that a longer follow-up duration could potentially **offer greater advantages** in thoroughly assessing efficacy and detecting effects on SC.’

P21: ‘… **the absence of a control group** restricts the ability to draw definitive conclusions about the efficacy of cERP compared to other treatment options or no treatment.’

The most frequent rebuttal in our study was about researchers claiming that more research was needed in order to strengthen, improve or add knowledge:

P18: ‘**further research**—most likely in laboratory settings— will… be useful to more fully understand these processes.’

P22: ‘Including comorbidities in **future analyses** could shed light on their influence on treatment outcomes and class transitions.’

In summary, we could say that the authors wrote about how credible their claims were, how much scope their claims had and how much insight or understanding researchers were able to get with their studies.

#### 3.4.2. Rebuttals of dissonant result claims

To rebut dissonant result claims, researchers used results that followed their expectations to support their expectations. This is the type ‘despite null, this other is significant’ in table 1:

P20: ‘This project found no consistent differences between inactive and nonspecific control groups. **However, all active treatments were significant** compared to TAU for all outcomes at posttreatment …’

P21: ‘**Although the lasso regression did not find this, the linear regression identified that** the item “To what extent did the patient cope with ERP tasks through leaning into the anxiety on day 3?” predicted the change in the Y-BOCS total score from baseline to post-assessment (β = 0.516, p = 0.050).’

As with consonant result claims, authors sometimes questioned the credibility of their dissonant results by mentioning potential alternative factors or methodological flaws:

P20: ‘… no treatment significantly reduced somatic symptom severity compared to WL (k = 20, n = 2 627, m = 22), **though the data on direct comparisons to WL was limited** (k = 3).’

P31: ‘We also prioritized observations that controlled for any other form of violence … However, in the interpretation of our findings, **it is important to consider that different types of GBV rarely occur independently.** Our estimates therefore probably represent the lower bound of risk experienced by an individual exposed to any of these forms of GBV, with co-occurrence leading to compounding health consequences.’

#### 3.4.3. Rebuttals of contested presuppositions

Researchers rebutted potential pitfalls that could put into question how trustworthy their study was in four ways. Firstly, authors attempted to rebut concerns about the presuppositions supporting the trustworthiness of their study and results by pointing to design choices, including multiple experiments or versions of it, that should either be preventive or protective against such concerns:

P23: ‘… the treatments were rigorously tested **under optimized, controlled conditions at a specialized treatment center** … Therefore, even though some noise was present, it is unlikely that different results would be observed if these treatments had been administered outside the research setting in a more natural environment.’

P37: ‘Notably, most participants in Study 1 provided only one or two descriptions of mental imagery. In response, **in Study 2, we refined the procedure** by instructing participants to generate and describe only the first mental image that came to mind in relation to risky activity.’

Secondly, the authors also refuted concerns about presuppositions using some kind of sensitivity analysis or other ways of checking them. This gain shows how results can be used in multiple ways to create arguments, for sensitivity analyses were used for backing, rebutting or drawing a claim.

P13: ‘… these ratings may differ from participants’ perceptions of AI capability when the study was conducted. Nevertheless, our meta-analysis **tested the moderating effect of publication year**, which accounts for perceived AI capability at the time when a study was published. Analyses found that publication year is not a significant moderator …’

P17: ‘There is a concern that individuals in the above-median group and the below-or-equal-median group are not balanced, undermining this study's identification strategy. **Therefore, balance checks were conducted across groups**…’

Thirdly, authors (partially) refute a concern about a presupposition with literature, pointing to a theory, past result(s), authoritative recommendation or rule of thumb that they had followed:

P25: ‘It is unclear if our findings apply to clinical samples with more comorbidity. However, promisingly, brief behavioral CBT-I interventions **are generally effective in samples of insomnia with mental comorbidity [58]** …’

P37: ‘Although the quality of data obtained from online labor markets has been questioned, **different analyses have suggested** that data collected on MTurk and Prolific Academic are valid and comparable to data collected via traditional methods (Buhrmester *et al.*, 2011; Mason & Suri, 2012; Peer *et al.*, 2017).’

Finally, while researchers acknowledge that the needed presuppositions to make their study trustworthy could be contested, they argued that the concerns were inconsequential or simply irrelevant to either the claims they were making with the study or the study itself:

P31: ‘Despite their inclusion, these data points are still minimal compared with the number of women-specific data points available, reflecting an imbalance in the existing data landscape that systematically overlooks GBV victimization as a health risk factor for men … **However, the consequences of GBV—regardless of the gender of the victim—are demonstrated by our present results**, even in the context of our highly conservative interpretation of the evidence available.’

P42: ‘… while we cannot entirely rule out the influence of specific task features in the present studies, **this explanation appears neither the simplest nor the most plausible**.’

## 4. Discussion

In this article, we have examined how researchers report their statistical reasoning in empirical research. We did so by inductively collecting qualitative data to create a partial typology of the types of statistical reasonings expressed in arguments within the larger context of scientific reasoning in papers. Our aim in examining 42 empirical studies in psychology was to explore rather than evaluate how researchers argue with and about statistics in the field.

We have distinguished different types of arguments in papers that share three distinct purposes: (i) give assurances about the legitimacy of inferences in the form of *backings*, (ii) make the movement from premises to claim in the form of *inferences*, and (iii) set constraints to claims in the form of *rebuttals*. These types of arguments could be applied in the context of consonant results, dissonant results or (credible or contested) presuppositions. In our results, we see two natures of statistical reasoning in empirical research. First, a sort of ‘legal’ nature of papers that may influence how arguments are presented. Second, the particularities that we see may be related to the nature of the reasoning itself.

### 4.1. The ‘legal’ nature of a paper in psychology

To begin with, when discussing inferences, we distinguished four types of arguments or reasonings in which researchers directly addressed their statistical result in order to make a claim: accepting the null hypothesis, rejecting the null hypothesis, estimation and description. Authors mostly reported the backing of their final argument, which would be the result of the test with or without its attached label; for example, the test is significant (*p* < 0.05), or the effect size is small (*d* = 0.2, 95% CI [0.01–0.03]).

With this, authors would claim that something was higher, lower, small, moderate, larger, different, the same, existing or absent based on a statistical result. This is closely related to the final claim of the study suggesting, for example, that a relationship exists between variables. In such reasoning, we contend that the report of the statistical result functions as a sort of *certification*. It certifies that a claim is empirically and statistically substantiated. It seems that this certification is the reason that the statistical report is intertwined with claims that are very similar to the study's conclusion: this is higher, this is reduced, there is no effect etcetera. Consequently, even though the backing and claims are reported, the statistical warrant remains implicit.

That is, a warrant like ‘if the probability of the observed data from an experiment or data more extreme occurring given that the claim is false is less than the established significance threshold (e.g. *p* < 0.05), the data are inconsistent with the claim (e.g. hypothesis, theory, etc.) being false’ is assumed to be known by the reader. Similarly, the estimation warrant was usually/always/typically left unspoken: ‘the relative frequency of observations of an event is fairly close to the event's probability of occurrence as the number of experiments is fairly large’. If manifested, authors would explicitly state the warrant with their result in the argumentation; for example, that the probability of the observed or more extreme data if the null hypothesis were true is below the significance threshold. Or that their observations lead them to believe that their 95% CI may include the true effect size, given that they have the property that if the procedure were to be repeated an infinite amount of times, it would contain the parameter in 95% of these cases.

The fact that scientific writing typically leaves the warrant implicit makes us believe that papers reflect a sort of ‘legal-like’ documentation procedure in which claims are justified or legitimized on how consistent the statistical proof is with the, frequently fuzzy, expectations, standards or certifying norms of the field or its subfields. These standards and expectations can be seen in an article in the form of, for example, the terminology accepted by our scientific community to certify or decertify a claim or its validity. We say that they are fuzzy because they usually do not show a clear meaning in papers; and yet, authors use them frequently, like when they use significance or an effect size label to characterize a result.

These procedures beget tacit argumentation where both the communicator and receiver assume that both know the behind-the-scenes statistical argument. That is, if a paper says that a finding is significant, it is assumed that the author and reader know what the implications are for the underlying hypotheses and expectations without further exposition. As we have mentioned in our introduction, this assumption is not substantiated by research, for in papers, surveys and textbooks mis- and over-interpretation of statistical concepts is frequent. Furthermore, in our research, we have seen authors erroneously claiming that the null was supported on the basis of statistically non-significant results (e.g. see [3]). This implicitness also happens with CIs and effect sizes. We found several instances of researchers using CIs as a backdoor to significance testing by stressing that the interval does not include zero. While it can be good practice to report estimated effect sizes, it does not add additional information if the final claim is written solely based on significance testing. Some may argue that this suffices because the goal is only to certify the potential presence of an effect. Nevertheless, under that logic, the reporting of the CI and the effect size still remain superfluous to that goal because the claim has nothing to do with the estimation paradigm.

To us, this ‘legal’ nature of a paper becomes evident in the strict compliance with the schematic segregation of sections within articles. In our study, we inspected the results, discussion, limitations and conclusion sections, which encompass the division promoted by the APA format [31]. Following the structure of these four sections preserves a certain sensation of order for the price of unification. When argumentation is unified, it looks different:

P14: ‘Finally, one should also keep in mind that, despite decades of research, the efficacy of existing treatments is still limited. The NNTs [Numbers Needed to Treat] we found in this meta-analysis exemplify this: even for the most effective interventions, compared to inactive controls, at least three patients need to receive the treatment to generate one additional case of improvement. It should be noted that this effect may still be overestimated. For most mental health problems, we found lower point estimates when only low-risk-of-bias studies were considered and maybe there are unknown biases that this analysis did not control for. We also saw that, for many indications, wait-lists still dominate as the comparator. As mentioned in the introduction, wait-lists are often considered one of the weakest control groups, and they might inflate treatment effects (Furukawa *et al.*, 2014; Mohr *et al.*, 2009, 2014). In the future, more realistic comparisons are needed to ensure that psychological treatments pass a “severe test” (Harrer *et al.*, 2023; Mayo, 2005).’

Here, the authors are integrating several reasons in favour of two claims, the first one being that the efficacy of current treatments seems to be limited. They say this *because* (i) they find that an NNT of 3 is still large when compared to inactive controls; *and* (ii) these effects might be overestimated *because* (ii.1) lower point estimates were found for low-risk of bias studies, *and* (ii.2) there might be other unknown biases; *moreover* (iii) the use of wait-lists as a comparison group also raises concerns about overestimation; *therefore*, (iv) better studies are needed – which is the second claim they are looking to make. You could agree or disagree with these two statements, but to do so, you would have to deal with the reasons the authors are giving for them. Reasons that are based on results that, in turn, should be held up by presuppositions that you may want to examine, too. A zealous attitude towards abiding by the segregation of information in APA style could obscure this argumentation, making it difficult to assess the reasoning's validity. We also must acknowledge that this integration reflects an expertise on meta-analysis besides psychotherapeutic research, so similar arguments with and about statistics may require some proficiency in statistics that many may not (want to) possess.

We can make a similar analysis of an integrated argumentation using our typology (note that integrated does not necessarily equal valid). Take for instance the next paragraph: ‘Our multilevel, multivariate meta-analysis provides the most comprehensive and rigorous compilation and assessment of relevant studies to date. We found a small effect in the expected direction, which remained robust to outliers and potential publication bias. At the same time, we found substantial effect heterogeneity. Moreover, in line with other researchers, we cannot preclude that the effect is in fact spurious or could even go in the opposite direction (as indicated by the prediction interval).’ The authors begin with (i) two backings—study characteristics—and, thus, their results; that is, doing a multilevel, multivariate meta-analysis, and then, with such methodology, providing the most comprehensive and rigorous compilation … to date. Later, they make (ii) an inference when they say that they found a small effect in the expected direction.

Then, the authors (iii) back again their result—consistency of sensitivity analyses—when they state that the effect remained robust to outliers and potential publication bias. They write (iv) a first rebuttal later: at the same time, we found substantial effect heterogeneity. Next, the authors back—literature—their study's high heterogeneity (v) in their phrase in line with other researchers, we cannot preclude that the effect is in fact spurious. We coded the part of the whole paragraph as (vi) a credibility rebuttal since the authors argue that their results could be owing to heterogeneity. Also, we coded the whole paragraph as well as (vii) a suggestive consonant result claim.

Our aim in this study was not to create a model of how argumentation is in empirical research, but rather to make an exploration of reasonings in research. To do the former, a much larger project is needed to hone, change, remove and add types in search of constructing a typological model. For exploration, we consider that our preliminary typology is enough.

Since we have fragmented language by coding in our study, it may be that the arguments that we have presented seem more fragmented than what they really are. Nevertheless, one of the reasons why we have not made an analysis of more complex arguments was that we did not find enough to group them into types.

We have argued that a paper can be seen as a ‘legal-like’ document in which statistical argumentation is used to settle a claim dispute. Heavily adapting [48, p. 7] the definition of argumentation, for us, statistical argumentation in empirical psychological papers is a written communicative act aimed at justifying knowledge or lack of it by putting forward a constellation of propositions and/or certifications the researcher can be held accountable for to make the alluded conclusions acceptable to the scientific community. While this is not detrimental *per se*, there might be so much focus on the social aspect of how papers should be written, what norms there are and what they should look like that it might cost space for reasoning and exposition (see also [49]). In the end, we can see the benefits of the social demands imposed by the scientific community that should improve the quality of the research output. Hence, the certifications of a paper that uses correlation could be the statistically significant result, mentioning that correlation is not causation as a limitation and having pre-registered the study as an assurance of the result. We would argue that although such certifications—since they follow standards of good research in our field—are useful, research needs reflection as it is more than a social endeavour.

It might be said that we are being too influenced by Toulmin's model of argumentation. After all, Toulmin got inspired by the legal setting and philosophy to develop his model. Since Toulmin developed it to create a layout to aid argument interpretation in real life, it could also be argued that the model fits the argumentation of statistical reasoning used in papers instead of the argumentation being accommodated into the model. We think that the layout has helped us to understand what was missing, for example the warrants, in our set of articles. It definitely is not the most up-to-date model [45], but it gave us a needed footing to analyse our data. It also provided a structure of what a consistent line of reasoning from the ground to the claim might look like. Consistency that is seen in the empirical endeavour as it begins with the setting of a hypothesis, its testing or estimation and the ensuing conclusion. In psychology, we attempt to establish warrants that could guide us in claiming something out of some other piece of information; for instance, providing therapy to someone suffering from depression could cause remission of symptoms. Psychology is a science that seeks to establish rather than disprove relationships. In that sense, the fact that Toulmin's model does not describe argumentations disproving or challenging a claim should not affect our understanding of the type of reasoning in papers that, instead, ought to be consistent to establish a warrant.

There are other alternative explanations for why researchers take shortcuts in their written statistical reasonings that do not have to do with our idea of certification. To begin with, researchers may have the desire to be concise; ‘authors seeking publication and students completing assignments increase their chances of success when they write concisely’ [31, p. 113]. It may also be the case that authors must be brief under journal norms. Three of the journals we examined (*American Psychologist*, *Journal of Applied Psychology* and *Nature Human Behaviour*) at the time of this writing have a word/page limit, two journals (*Cognition* and *Psychotherapy and Psychosomatics*) had recommendations about succinctness, while *Psychological Bulletin* was the only one that did not have an explicit statement about it. Finally, it may also be the case that researchers assume that their audience shares specialized knowledge with them. This assumption is bona fide, for researchers do write for quite specific, small audiences. Yet, it also derives from the idea that writing an article is mainly about telling or transmitting knowledge [50,51]. While assuming that the audience knows or already understands what one is talking about is slightly different than what we have deemed here as certification, they both may come from the same philosophical tradition underpinning scientific writing in psychology papers:

…readers are no longer cast in the role of people trying to understand or solve some problem. Rather they are presumed to be looking for additional bits of knowledge to fit in with their previous bits … The author must display competence to the audience, rather than persuade readers of the truth of an idea. If properly demonstrated by a-proper experiment, the hypothesis must be accepted by the audience. In an intellectual sense, the audience has little to say about the meaning of an experiment or even about the truth of a hypothesis. Its role, rather, is to judge the propriety of the experimental proof. [52, pp. 274–275]

### 4.2. The particular nature of statistical reasoning in empirical research

While statistics is a deductive, logical structure from which conclusions can be drawn [24], we believe that in empirical research statistical reasoning takes the form of defeasible reasoning, for ‘… the premises taken by themselves may justify us in accepting the conclusion, but when additional information is added, that conclusion may no longer be justified’ [53, p. 481]. Particularly, the presence of rebuttals shows this nature of applied statistics. Once we work with empirical data, there will always be a ‘but’ in our experiment. Something will not go according to plan, subpopulations will be left out, a type I or II error might occur, or a variable will not be taken into account. There will always be questions and challenges to claims hinging on collected data because new data could always change those claims. In psychology, it is even more important to recognize that because we are dealing with human behaviour, we will be guaranteed not to do it perfectly in every circumstance and context.

This brings about, first, the importance of attaching qualifiers of uncertainty to our claims. We need to deal with the fact that the idealized experiment does not exist, and, hence, full certainty cannot be part of empirical research. Even if multiple studies are conducted, there are still some beliefs that we as researchers depend on, yet they are beliefs nonetheless. For example, regularity in nature—whatever has happened in the past will happen in the future—in our research is an assumption with which researchers made inferences. However, ultimately, nothing really guarantees that what happened yesterday will happen again tomorrow, let alone in human behaviour.

Regarding this belief, it was not always clear to us how researchers claimed that there was consistency between their results and others besides being statistically significant or showing effect sizes being superior to a comparison group. Moreover, sometimes, consistency was claimed without any explicit warrant or backing. Hence, it may also be important to define, as a community, to what extent we expect consistency among multiple studies or results. For instance, should researchers expect all the experiments they conduct within a single study to be significant? If we, as a community, cannot adjudicate a final answer on this, then we should equip researchers with the thinking tools to adjudicate this themselves in their study. After all, thinking tools should be essential to deal with the inherent inconsistencies—rebuttals—of research. In our, as a community, idealized vision of science, things may be too consistent. This was evident when we saw that researchers were even using non-significant results to support the consistency of their hypotheses and research.

Statistical reasoning in empirical research in psychology is particularly/unavoidably subjective. Researchers apply a deductive structure of reasoning to a concrete, contextualized moment and from a particular point of view. Let us use an example to clarify this point [54]. To start off, let us say that I want to sit down, and I see a chair in the distance. When I get close to that chair, I see that it is weak, so I do not believe that it will hold me up and look for another chair.

We can connect from this point on the continuation of the example with what happens in research. In science, we get closer to a phenomenon, examine it and give reasons for students, researchers and practitioners to believe whether a theory, description or intervention will hold up or not. We build knowledge about the sturdiness of the chair by adducing reasons like its materials, usage and dimensions. In the end, we can claim that a chair is weak, yet when we sit on it, it turns out to be strong and remains firm despite what we said. Or claim that it is sturdy, only to fall over after sitting down because the chair is weak. The job of the scientist is to give reasons, with their inherent uncertainty, to believe in the sturdiness or weakness of the chair. Whether the chair holds up does not depend on the researcher's explanation, but the knowledge that it will do so does depend on how close that explanation, with its respective arguments, is to the truth about the chair's sturdiness [55]. For example, the first author will never forget how he fell at a conference because one of the legs of his plastic chair had bent owing to sunlight that had become stronger because it was passing through the thick glass of a window for hours—this happened to nobody else, for their plastic chairs were quite safe under different conditions. Therefore, with our explanations, we must get as close as possible to the truth, whatever that is, of the phenomenon, but in the end, there will always be uncertainty about whether the chair holds up or not. It might happen that some other variable, a different condition or any type of additional information was not taken into account by our explanation, and that we might have to change our knowledge and beliefs about the phenomenon.

This example is also relevant for explaining why, in our research, literature or sensitivity analyses could be used in multiple ways for different reasons. An author must write a valid argumentation, but there is not one way to do it. Statistics is so vast and complex that we cannot imagine an adequate use and approach to it that sloughs off reflection and rationality. Even in the group of papers that we have analysed, there was a smorgasbord of approaches, and in the effort to find patterns, we might have lost nuance in describing our results in the end. Namely, arguments may be more complex than what we are depicting here, not only outside of our sample, but within it as well. In addition, to balance the handling of all the papers, we gathered data related to the first inferential claim rather than collecting data from multiple results, which could have affected our analysis. This is merely a first step to finding out how researchers write arguments with and about statistics to, then, elucidate how their reasoning is.

In summary, we believe that argumentation in empirical research must be logical, clear and reasonable if we accept the role of the scientist as the one who gives reasons for belief. This also implies that when researchers see a weak chair, they denounce it. Therefore, a reasonable argument must be accompanied by uncertainty and transparency. Perhaps it might be time to question whether the way we write research in psychology conforms to these principles.

## 5. Research transparency

Our study's goals, design and analytical approach were pre-registered on the Open Science Framework (OSF) [56]. We made the following deviations to our original preregistration:

— We originally planned to use only content analysis. In a later stage, we added the construction of a typology which employed elements from Toulmin's model, because we became aware of this and believed it to be very suitable in classifying researcher's arguments.
— We replaced the journal *Trends in Cognitive Sciences* with *Cognition*, because the former publishes few empirical articles.
— We initially planned to evaluate the validity of statistical arguments, but we changed our approach to a more neutral one, so as to remain scientifically dispassionate.

## Supplementary Material



## Data Availability

Our full dataset is available on the OSF repository [57]. Supplementary material is available online [58].

## Footnotes

^1^
Since *Nature* had many eligible articles, we generated a random sequence in R to pick them.

^2^
The P and number before each quotation indicates from what document the quotation is, so P1 would be the first paper we reviewed.
