# Supplementary material for: Types of statistical arguments in empirical research in psychology
Source: R Soc Open Sci. 2026 Oct 28;13(10):260798. doi: 10.1098/rsos.260798 (PMC13714387; doi:10.1098/rsos.260798)
Supplement: Supplementary file 1 [file rsos.260798.zip › rsos.260798/rsos260798_si_002.docx]

**Appendix B**

*18 logical relationships used in Arcing*

| Type | | Name / Symbol | Definition | Some Connecting Keywords |
| --- | --- | --- | --- | --- |
| Coordinate Relationships | One proposition does not support the other, but each makes its own contribution in the whole | Series (S) | Each proposition makes its own independent contribution to the whole. | And, moreover, likewise, furthermore, likewise, neither, none, nor, etc. |
|  |  | Progression (P) | Like series, but each proposition is a further step toward a climax. | Then, and, moreover, furthermore, etc. |
|  |  | Alternative (A) | Each proposition expresses a different possibility arising from a situation. | Or, but, while, on the other hand, etc. |
| Subordinate Relationships | Support by restatement | Action-Manner (Ac/Mn) | The statement of an action, and then a more precise statement which indicates the way or manner in which this action is carried out. This relationship can also be used to indicate means. | In that, by, participles, etc. |
|  |  | Comparison (Cf) | An action and a statement that clarifies that action by showing what it is like. | Even as, as… so, like, just as. |
|  |  | Negative-Positive (-/+) | The relationship between two alternatives, one of which is denied so that the other is enforced. This is also the relationship implicit in contrasting statements. | Not… but. |
|  |  | Idea-Explanation (Id/Exp) | The relationship between an original statement and one clarifying its meaning. The clarifying proposition may expound on only one word of the previous or other propositions. | That is. |
|  |  | Question-Answer (Q/A) | The statement of a question and the answer to that question. | (look for the question mark) |
|  | Support by distinct statement | Ground (G) | A statement and the argument or reason for the statement (supporting proposition follows) | For, because, since, etc. |
|  |  | Inference (I) | A statement and the argument or reason for the statement (supporting proposition precedes) | Therefore, wherefore, consequently, accordingly, etc. |
|  |  | Bilateral (BL) | A bilateral proposition supports two other propositions, one preceding and one following. | See conjunctions for Ground and Inference. |
|  |  | Action-Result (Ac/Res) | An action and a consequence or result which accompanies that action. | So that, that, with the result that. |
|  |  | Action-Purpose (Ac/Pur) | An action and its intended result. | In order that, so that, that, with a view to, to the end that, lest. |
|  |  | Conditional (If/Th) | Like Action-Result except that the existence of the action is only potential and the result is contingent upon that action. | If… then, provided that, except, etc. |
|  |  | Temporal (T) | A statement and the occasion when it is true or can occur. | When, whenever, after, before, etc. |
|  |  | Locative (L) | A statement and the place where it is true or can occur. | Where, wherever, etc. |
|  | Support by contrary Statement | Concessive (Csv) | A main clause that stands despite a contrary statement. | Although, yet, though, nevertheless, but, however. |
|  |  | Situation-Response (Sit/R) | A situation and its surprising or counter-intuitive response. | And. |

*Note.* This table is adapted. To see more complete tables, go to Piper (1999) and Bethlehem College & Seminary (2025).
