# Supplementary material for: Types of statistical arguments in empirical research in psychology
Source: R Soc Open Sci. 2026 Oct 28;13(10):260798. doi: 10.1098/rsos.260798 (PMC13714387; doi:10.1098/rsos.260798)
Supplement: Supplementary file 1 [file rsos.260798.zip › rsos.260798/rsos260798_si_003.docx]

**Appendix C**

*Quotations for each type of statistical argument in empirical research papers*

| Combination of Attributes | Type of Argument | Quotations |
| --- | --- | --- |
| Backing Consonant Result Claim | Literature | P9: “We **build upon prior** meta-analyses indicating that reduced memory specificity is…, by demonstrating that memory specificity is a predictor of disorder onset.”  P15: “We found that the effects of therapy were…, **which is in line with previous research** (Cuijpers, Karyotaki, Reijnders, Purgato, & Barbui, 2018).”  P22: “This result **aligns with existing literature** on therapeutic interventions for….”  P27: “Our findings focusing on Europe **mirror** somewhat those of Surkalim et al. (2022).”  P39: “**In line with this finding, previous studies have shown** that episodic RCJs of older adults depend on...” |
|  | Consistency of Sensitivity Analyses | P8: “Importantly, all relationships **remained** **significant after controlling for** previously examined moderators (leader benevolence and power distance orientation) and alternative positive and negative states”  P13: “All results **remained robust** **under this alternative categorization.**”  P26: “The results of the prescribing psychologist versus psychiatrist analysis **were largely consistent in the sensitivity analyses**.”  P31: “our one-star association **was consistent across all the sensitivity analyses** for this outcome...”  P36: “These results were **supported by the analyses** on pain unpleasantness ratings (see APPENDIX A: Supplementary material 3).” |
|  | Study Characteristics | P1: “**With this comprehensive multilevel, multivariate meta-analysis**, we set out to shed light on the question…”  P3: “**Using data collected from field and experimental studies**, we find that…”  P6: “**Using bootstrapping techniques** (Cheung & Lau, 2008), I find that the indirect effect of…”  P9: “This **preregistered** IPD-MA evaluated whether…”  P10: “In **the first preregistered hypothesis** it was predicted that the investigated conditions would... This hypothesis was confirmed…” |
|  | Explanation | P1: “which would be in line with **previous theorizing that** people from more collectivistic societies are more prone to intuitive deontology (e.g., Greene, 2008).”  P7: “...we obtained this pattern of results even though the description of the actual transgression was kept constant across conditions. **That is**, …[t]he only difference across conditions in these studies was the framing label for the transgression.”  P9: “There was very little difference in the risk associated with moderate or high-memory specificity, **consistent with the theory** (Williams et al., 2007) that mental illness only arises if...”  P21: “These day-specific changes align with the structured design of B4DT, **in which each day of the treatment is intended to** target different psychological processes.”  P42: “...the analyses revealed a Prime valence X Movement interaction effect, t(210.8) = 4.60, p < .001, CI [0.055; 0.14], dz = 0.32, **consistent with** the AA compatibility effect.” |
| Backing Dissonant Result Claim | Despite small, the effect is important | P11: “**Although** **overall effect sizes were small-to-moderate,** **small effects are clinically meaningful** when accumulating over time (Funder & Ozer, 2019).”  P12: “**Small but significant** differences across levels of cognitive demand (p = .005) emerged for chronic studies”  P41: “...we also observed **a slight, albeit significant**, drop in memory accuracy with longer delay durations.” |
|  | Literature | P1: “Moreover, **in line with other researchers**, we cannot preclude that the effect is in fact spurious...”  P12: “The overall small positive effects of… chronic (g = 0.25) [is] **consistent with**… (Álvarez-Bueno et al., 2017; Ludyga et al., 2020)... Instead, our small positive effect for chronic studies is in contrast with the negligible effect reported by Ciria et al. (2023)...”  P27: “Our findings for midlife in the United States contrast with those of historical declines and stability observed in… (Clark et al., 2015; Hawkley et al., 2019; Mund et al., 2020) **but are similar to**… (Buecker et al., 2021)”  P34: “This finding **is in line with other international studies** on trust in scientists 5–7. Our study thus confirms, expands and strengthens previous work that refutes the narrative of a wide-ranging crisis of trust”  P38: “...the effect of outcome processing on the SoC ratings was much higher than the P300 effect, **in line with the previous studies** involving neuroimaging methods, sense of control, and agency (El Zein et al., 2022; Sidarus et al., 2017).” |
| Backing Credible Presuppositions | Avoiding Type III Errors | P7: “**To test our specific hypotheses, we intentionally chose not to include** contextual information...”  P12: “The focus of the present review was… on the role of a range of moderators, including the qualitative characteristics of physical activity. **To this aim, we also included** physical activity interventions…”  P29: “**To confirm this, we created a** comparison group for 2,743 retracted authors by matching them with a non-retracted author, as leaving scientific publishing can be a result of many reasons other than retraction.”  P31: “...the included male-specific observations are narrowly focused on intimate partner physical violence against men **to reflect this study’s emphasis on** gender-based physical violence”  P42: "**Consistent with the informational accounts of the AA compatibility effect** (Pillaud & Ric, 2022), participants [sic] instructed to ask themselves whether they should approach the target exhibited an AA compatibility effect whereas this effect was reversed when participants were instructed to ask themselves whether they should avoid" |
|  | Literature | P12: “We maintained those outliers **in line with Viechtbauer and Cheung (2010)**...”  P8: “We then **followed Aiken and West’s (1991) suggestion to** plot the interaction effects at high (+1 SD) and low (−1 SD) levels of event disruption...”  P13: “**Following previous meta-analyses** (Dai et al., 2023; Donald et al., 2021; Macnamara & Burgoyne, 2023), we assessed study quality based on four indicators...”  P22: “However, **as a rule of thumb**, the profile should be rejected if it includes <3.0% of the total sample size… [52, 53].”  P24: “...a two-sided 95% confidence interval (CI) was above the non-inferiority limit set at −3. The limit of 3 **was chosen based on previous noninferiority studies** on OCD [41].” |
|  | Design | P12: “It is important to note that the adult population (n = 127) **was equally represented** compared to children and adolescents (n = 112)”  P24: “**The primary strengths of this trial** include its robust RCT design, in which we rated adherence to both treatment manuals, included long-term follow-up, and conducted the trial in real-world clinical settings.”  P44: “We used **multilevel meta-models with clustered standard errors at the sample level to account for** cases in which the same sample contributed various effect sizes (that is, the meta-analytic units of observation).” |
|  | Statistical Bedrock | P9: “Low heterogeneity supports the consistency across studies and **lends confidence** to results.”  P10: “Moreover, the overall effects of each updated meta-analysis were the product of an omnibus analysis (and not of 10 separate meta-analyses), **protecting against** Type 1 errors.”  P16: “Throughout the sequential model specifications, the ALD models that included a cubic term **always fit the data better**, providing some evidence of nonlinear SEC growth patterns as students move through school”  P26: “The results of our models and **the assumption test** demonstrating that prescribing psychologists and psychiatrists are statistically similar alternatives (i.e., failed independence of irrelevant alternatives test) provide evidence that prescribing psychologists are indeed treating a patient population similar to that of psychiatrists”  P36: “The quantitative model comparison using **the Bayesian information criterion (BIC) affirmed that** the Bayesian surprise model outperformed the Bayesian estimate model (Fig. 3d).” |
| Inferences and Consonant Result Claims | Accepting the Null Hypothesis | P2: “...the relationship was strong and significant for liberal participants (slope = −.64, p < .001) but nonsignificant for conservative participants (slope = −.21, ns)... as I predicted, only liberal employees tend to view their politically dissimilar coworkers as lower in person trustworthiness; **the pattern does not appear among conservatives**.”  P8: “...there was **no difference in anxiety** across the high (M = 2.06, SD = 0.89) and low (M = 1.82, SD = 0.83) authoritarian leadership conditions, F(1, 182) = 1.02, p = .314, η2 = .01.”  P17: “In summary, it was found that **none of the treatments alone affected** individual cooperation with stay-home activities. However, a significant effect was found when they were combined.”  P23: “Remarkably, EMDR-only and EMDR-DBT **did not differ** in their reduction of PTSD symptoms…”  P39: “There was **no quadratic effect** in either episodic (age-squared beta mean: -0.04; HDI: [− 0.13; 0.04]) or semantic tasks (age-squared beta mean: 0.03; HDI: [− 0.05; 0.11])” |
|  | (Not-)Rejecting the Null Hypothesis | P3: “...**the relationship was significant** (γ02 − γ60 = −.56, p < .001; Model 3; Table 4). Hypothesis 1 was thus supported.”  P6: “I also found **a significant positive relationship** between experimentation T4 and BM adaptation T5 (b = .172, SE = .052; p = .001).”  P9: “Reduced memory specificity **significantly increased odds of** a future diagnosis of… and **significantly reduced** **the time** to first onset of that disorder.”  P12: “Core executive function (g = 0.16, 95% CI [0.08, 0.24]), higher level executive function (g = 0.28, 95% CI [0.18, 0.39]), and nonexecutive cognitive function (g = 0.32, 95% CI [0.16, 0.48]) had small ES **with CI not including zero**.”  P20: “Further, Egger’s test was not significant (p = 0.35), i.e., **not indicating publication bias** for the whole network.” |
|  | Estimation | P11: “effects from those studies (b = 0.04 [−0.02, 0.09], n = 169,300, K = 78) **did not differ from those** that used less appropriate measures (e.g., listwise deletion; b = 0.09 [0.01, 0.17], n = 41,314, K = 41).”  P12: “...physical activity had **a small positive effect on cognition** (g = 0.25, 95% CI [0.19, 0.31], k = 171) with considerable heterogeneity (I^2^ = 89.8%).”  P14: “**The largest effects were yielded by treatments for** OCD, PTSD, and specific phobias (g = 1.18–1.25; NNT: 4–3).”  P21: “All subscales changed over the 4 concentrated treatment days **with small to medium effect sizes** (0.12–0.66)”  P26: “Insomnia was the only physical diagnosis associated with **an increased probability** of seeing a specialist (AME = 0.023, CI [0.004, 0.042])” |
|  | Description | P13: “A 3D figure (**Figure 4**) visualizing the full distribution of effect sizes as a function of the two dimensions of the Capability–Personalization Framework further supports the framework...”  P16: “**As indicated by Figure 1**, results for GM generally showed increases in scores over time...”  P21: “First, the high rates of adherence to ERP strategies, **with compliance above 70% on day 2 and above 80% on day 3**, indicate that patients generally maintained a strong commitment to the therapeutic tasks throughout the intervention.”  P29: “We find, **descriptively**, that authors whose papers were retracted due to misconduct and plagiarism are more likely to…”  P40: “We found that accuracy and confidence gradually decreased after the start of both learning and recognition blocks and reset in the recognition block (**Fig. 1k**).” |
|  | Regularity | P3: “The findings of the field surveys and experiments **collectively demonstrated** that…”  P4: “We **replicated our findings** in Singapore, an Eastern culture with distinct parent–child relationships.”  P5: “In Study 1, while using supervisors’ reports of higher level managers’ leadership styles provided a separate rating source that helps to reduce common methods bias, those ratings may not align with employees’ perceptions of the managers. Yet, **consistent results from Study 3 boost confidence in our findings**.”  P7: “...**obtaining the same pattern of results** for two very different kinds of ethical transgressions (i.e., bribery and fraud) and several different euphemisms for each transgression suggests that these euphemism effects are not merely the result of an [sic] something specific to an idiosyncratic case.”  P10: “This hypothesis was confirmed by **the statistical analyses that clearly showed** that the overall case–control effect was not independent of diagnoses.” |
|  | The Study | P8: “...**this study provides** preliminary support for our theoretical model…”  P20: “**This project found no** consistent differences between inactive and nonspecific control groups.”  P21: “**The present study provides significant insights** into brief cERP for OCD – specifically the B4DT – revealing several key findings that contribute to a better understanding of the mechanisms behind cERP.”  P26: “**This study provides a foundational understanding** of care delivered by prescribing psychologists versus other health care providers”  P34: “**Our study thus confirms, expands and strengthens** previous work that refutes the narrative of a wide-ranging crisis of trust.” |
|  | Suggestive Results | P10: “Although our results do not provide causal and mechanistic insight, **they nonetheless suggest that** atypical hand preferences are...”  P12: “Importantly, some evidence consistent with risk of publication bias emerged for chronic studies more pronouncedly than for acute studies. This evidence calls for **caution when drawing conclusions**.”  P14: “It should be noted that **this effect may still be overestimated**. For most mental health problems, we found lower point estimates when only low-risk-of-bias studies were considered and maybe there are unknown biases that this analysis did not control for.”  P15: “...despite these findings, we should consider this data set highly heterogeneous, which may indicate underlying differences between trials that we did not identify. The results should **be considered with caution**.”  P31: “**While we did not have enough men-specific data** for anxiety disorders to run a separate sensitivity analysis just for men, there was insufficient evidence of a statistically significant association between physical GBV and major depressive disorder when restricting the data only to men...” |
| Inferences and Dissonant Result Claims | Embracing Unexpected Results | P8: “Although the results… provide a convergent pattern of support for our theoretical model, **some inconsistent findings prompted us to explore** their possible reasons and implications.”  P16: “...by age 12, students typically realize that sometimes ability can limit outcomes, regardless of the amount of effort. Therefore, **it is surprising that** GM continues to rise through adolescence.”  P17: “**The first striking result of the experiment** was that no strong support for the main effects of a powerful messenger was found, as shown in Rows 5–8. This result is in sharp contrast to the results of previous studies...”  P26: “**This is in direct contrast to** a survey of prescribing psychologists in which 40% of respondents reported that they were treating more patients from rural areas (Linda & McGrath, 2017). It is plausible that prescribing psychologists see fewer patients from rural areas than urban areas but see more patients from rural areas than they did before becoming prescribers.”  P39: “Indeed, our results suggested that metacognitive efficiency related to a semantic task increased with age. Previous studies showed no effect of age on semantic metamemory (Dahl et al., 2009; Eakin et al., 2014; Kav´e & Halamish, 2015;Marqui´e & Huet, 2000; Morson et al., 2015; Souchay et al., 2007)... Thus, **the limitations of the metacognitive measures used in previous studies** (i.e., gamma index) may not have allowed to highlight the positive effect of age on semantic metamemory.” |
|  | Despite significant, it is meaningless | P:30 “...effect sizes were positive, statistically significant **but equivalent** (that is, they fell within the preregistered SESOIs). This indicates a difference that is too small to be theoretically meaningful between adolescents with and without a mental health condition.”  P31: “We identified an association between physical GBV and seven health outcomes…**although these associations and/or their underlying evidence were rated as weak**, receiving one- and two-star ratings in the Burden of Proof framework.” |
| Rebuttals of Consonant Result Claims | Credibility | P6: “Conducted at the start of the pandemic, it employed a program for entrepreneurs, which **may have influenced** my findings.”  P11: “However, longitudinal studies can still be subject to confounding. It is possible that **a third variable explains both** socioemotional problems and screen use.”  P17: “The effect size for this comparison was 4317 min, which is a substantial magnitude given the control mean value. **However, this estimate was not significant** (p = .153).”  P19: “Second, the study sample size is small; thus, **we cannot be certain that** some beneficial and adverse effects might have been missed (type II errors).”  P26: “Furthermore, **the availability of training programs may have limited the rollout** of RxP in Louisiana during the first few years of their policy, perhaps resulting in the early years of the study period primarily reflecting patients from New Mexico.” |
|  | External validity | P2: “Nonetheless, my findings **may not generalize** across the globe.”  P6: “However, they **may not be universally generalizable**. Self-promotion in more traditional settings is less likely to provide the agentic benefits I have theorized.”  P13: “...all studies in our meta-analysis were conducted before the launch of ChatGPT in November 2022. Given the revolutionary nature of ChatGPT and other generative AI models, people’s perceptions of AI capability **may have evolved** **over time**.”  P23: “...the lack of gender diversity in our sample, predominantly consisting of females, **restricts the generalizability** of our findings to other genders.”  P25: “It is unclear if our findings **apply to** clinical samples with more comorbidity.” |
|  | Non-experimental design | P11: “Without using experimental designs or more sophisticated causal models, **we cannot rule out** possible confounds...”  P13: “...we emphasize that meta-analysis is primarily a correlational method, particularly concerning the moderator analyses. Hence, the results reported in our meta-analysis **do not indicate** causal relations.”  P18: “A significant limitation of our study is that we rely on observational data, and we are therefore **unable to make any strong** causal claims.”  P30: “...we analysed raw associations from cross-sectional data. Therefore, no causal or directional inference **can be drawn from** these findings...”  P40: “Our findings related to the functional consequences of gaze dynamics **are observational** since we did not experimentally manipulate gaze dynamics.” |
|  | Design | P9: “There was considerable **variability in the timing of follow-up assessments** between disorder types (e.g., latest assessment of 8 months for PTSD vs. 48 months for depression)”  P15: “Second, **we only examined** short-term effects and depressive symptoms as outcomes.”  P19: “We posit that a longer follow-up duration could potentially **offer greater advantages** in thoroughly assessing efficacy and detecting effects on SC.”  P21: “...**the absence of a control group** restricts the ability to draw definitive conclusions about the efficacy of cERP compared to other treatment options or no treatment.”  P38: “...**our design did not include** all potential determinants of human rationality to be tested within the context of the sense of agency.” |
|  | Future research | P5: “Moreover, while our performance outcomes cover three major forms of job performance, **future research** may consider additional outcomes influenced by exhaustion, such as creativity and proactive behaviors.”  P7: “**Future work** would do well to test other possible mediators for these effects.”  P18: “**further research**—most likely in laboratory settings— will… be useful to more fully understand these processes.”  P22: “Including comorbidities in **future analyses** could shed light on their influence on treatment outcomes and class transitions.”  P25: “Still, the (cost-)effectiveness of telephone-guided SRT **will need to be tested** in clinical settings.” |
| Rebuttals of Dissonant Result Claims | Despite null, another one is significant | P20: “This project found no consistent differences between inactive and nonspecific control groups. **However, all active treatments were significant** compared to TAU for all outcomes at posttreatment...”  P21: “**Although the lasso regression did not find this, the linear regression identified that** the item “To what extent did the patient cope with ERP tasks through leaning into the anxiety on day 3?” predicted the change in the Y-BOCS total score from baseline to post-assessment (β = 0.516, p = 0.050).”  P24: “When only the baseline measure was included as a covariate, the 95% CI for OCD symptoms was just over the specified noninferiority margin of 3 (95% CI: −3.19; 1.43). **When the remaining covariates were included, the 95% CI was below the margin** (95% CI: −2.74; 1.97). Thus, ACT was interpreted to be non-inferior to CBT treatment.”  P28: “...the 26% dropout rate from the present study was somewhat lower than the meta-analytic dropout rate of 36% among OEF/OIF/OND veterans seeking PTSD treatment (k = 20; Goetter et al., 2015). When the present meta-analysis estimated dropout among similar samples (OEF/OIF/OND era only, PTSD treatments, and VA settings) as Goetter and colleagues’ meta-analysis, **the dropout rate was then 40%**.”  P34: “While trust in science is moderately high overall, there are notable variations across countries and regions (Fig. 1). Contrary to previous studies [...] , we did not find a clear pattern that scientists are less trusted in Latin American and African countries. **However, we did find patterns** **within specific regions**. For example, Russia...” |
|  | Credibility | P1: “Most of the studies included in this meta-analysis were conducted in WEIRD and individualistic societies (**a shortcoming also identified in other meta-analyses in the field**, see Rehren, 2022). Therefore, the role of culture for determining intuitive deontology could not be adequately addressed and should be studied in subsequent research projects”  P10: “...there could also be another explanation as to why the meta-analysis on mixed-hand preferences showed a contrasting result pattern. Even though categorizing individuals into left- and right-handers is usually straightforward, **the criteria for categorizing** individuals as mixed-handers are often inconsistent between studies. Mixed-handedness may reflect ambidexterity… or inconsistent hand use…”  P19: “...contrary to our initial hypothesis, we did not observe significant changes indicating improvement in ToM from the intervention… both aspects should be considered when developing new training programs or assessment tools, **a consideration that this study did not address**.”  P20: “...no treatment significantly reduced somatic symptom severity compared to WL (k = 20, n = 2,627, m = 22), **though the data on direct comparisons to WL was limited** (k = 3).”  P31: “We also prioritized observations that controlled for any other form of violence… However, in the interpretation of our findings, **it is important to consider that different types of GBV rarely occur independently.** Our estimates therefore probably represent the lower bound of risk experienced by an individual exposed to any of these forms of GBV, with co-occurrence leading to compounding health consequences.” |
| Rebuttals of Contested Pressupositions | Design | P23: “...the treatments were rigorously tested **under optimized, controlled conditions at a specialized treatment center**… Therefore, even though some noise was present, it is unlikely that different results would be observed if these treatments had been administered outside the research setting in a more natural environment.”  P37: “Notably, most participants in Study 1 provided only one or two descriptions of mental imagery. In response, **in Study 2, we refined the procedure** by instructing participants to generate and describe only the first mental image that came to mind in relation to risky activity.”  P30: “Second, all participants in the sample underwent a standardized multi-informant assessment by professional clinical raters. **This method provided** **comprehensive information** about participants’ mental health without only relying on self-reported questionnaires, self-diagnoses or mental health service use, measures that only capture a subset of individuals with mental health conditions.”  P41: “Given these considerations, **we ran two versions of the task**: one with randomised and thus unpredictable delay durations (Experiment 1), and another with blocked and therefore predictable delay durations (Experiment 2)... This underscores that the mechanisms underlying spatial organisation in working memory, as reported here, are robust and not dependent on the predictability of delay durations.”  P42: “Such a sequential presentation of the prime and the target may have paradoxically encouraged participants to unintentionally use this information to anticipate their reaction toward the target. To rule out this explanation, **we conducted a second experiment**...” |
|  | Sensitivity Analyses | P9: “**Missing diagnostic outcome data did not significantly (ps > .05) correlate** with participant’s age (r = −.03), sex (r = −.03), or baseline memory specificity (r = .06).”  P13: “...these ratings may differ from participants’ perceptions of AI capability when the study was conducted. Nevertheless, our meta-analysis **tested the moderating effect of publication year**, which accounts for perceived AI capability at the time when a study was published. Analyses found that publication year is not a significant moderator...”  P17: “There is a concern that individuals in the above-median group and the below-or-equal-median group are not balanced, undermining this study’s identification strategy. **Therefore, balance checks were conducted across groups**...”  P18: “The three emotions show substantial intercorrelations, meaning that introducing them into the same equation can be problematic since it may lead to issues of multicollinearity and potential suppression effects (cf.Jost, 2019). Nevertheless, as Supplemental Table S2 shows, all three emotions remained strongly **positive and statistically different from zero even when added into a regression simultaneously**.”  P26: “First, we had to rely on an algorithmic approach to identifying prescribers due to the absence of prescriber information... This limitation warrants significant consideration, as prescriber identification was a critical component of the present study; however… **the consistency of our results across sensitivity analyses** using different identification methods instill confidence that our results are robust to misclassification related to this limitation” |
|  | Literature | P22: “We recognize that this study is exploratory, indicating that certain classes may have a limited number of participants. However, **as a rule of thumb**, the profile should be rejected if it includes <3.0% of the total sample size, [sic] the profile should be rejected [52, 53]”  P25: “It is unclear if our findings apply to clinical samples with more comorbidity. However, promisingly, brief behavioral CBT-I interventions **are generally effective in samples of insomnia with mental comorbidity** **[58]**...”  P26: “It is possible that the timing with which private insurance began to cover prescribing psychologists may result in their patients being underrepresented in the earlier years of this study. However, **a prior study found** that private insurance is one of the major reimbursement sources for prescribing psychologists (Peck et al., 2021), suggesting that this limitation is not cause for concern.”  P37: “Although the quality of data obtained from online labor markets has been questioned, **different analyses have suggested** that data collected on MTurk and Prolific Academic are valid and comparable to data collected via traditional methods (Buhrmester et al., 2011; Mason & Suri, 2012; Peer et al., 2017).”  P42: “...it remains possible that evaluation directly triggers other behavioral manifestations of approach and avoidance, which are not captured by instruction-based behavioral tasks, such as the VAAST or the Manikin task (e.g., Fini et al., 2020)... However, it should be noted that the VAAST (as well as the Manikin task) were designed to capture phenomenological aspects that occur during the execution of AA movements, and that **these tasks have provided some of the strongest and the most easily replicable AA compatibility effects**.” |
|  | Inconsequential | P12: “Our data showed a substantial to considerable heterogeneity, but the results were not adjusted for heterogeneity, because **we did not aim at** testing overall effects of physical activity on cognition but the individual and joint role of a prespecified set of moderators that were expected to explain the presence of heterogeneity (Johnson, 2021).”  P21: “the absence of a control group restricts the ability to draw definitive conclusions about the efficacy of cERP compared to other treatment options or no treatment. However, **given that this study was a preliminary investigation, its primary aim** was to assess the implementation of the cERP within the German healthcare system [14].”  P28: “...there are likely articles providing a dropout rate for service members and veterans that were not included in this study. Although this meta-analysis aimed to be as comprehensive as possible, **the primary objective of this study** was to obtain a representational sample of articles reflecting the literature conducted to date…”  P31: “Despite their inclusion, these data points are still minimal compared with the number of women-specific data points available, reflecting an imbalance in the existing data landscape that systematically overlooks GBV victimization as a health risk factor for men [...]. **However, the consequences of GBV—regardless of the gender of the victim—are demonstrated by our present results**, even in the context of our highly conservative interpretation of the evidence available.”  P42: “...while we cannot entirely rule out the influence of specific task features in the present studies, **this explanation appears neither the simplest nor the most plausible**.” |

*Note*. Papers P32, P33, and P35 were excluded from our data because we did not find their complete versions. They were replaced with P43, P44, and P45. The sample size, then, remains the same; 42 papers in total.
